# Adherence to Coronavirus Disease 2019 Preventive Measures in a Representative Sample of the Population of the Canton of Vaud, Switzerland

**DOI:** 10.3389/ijph.2022.1605048

**Published:** 2022-08-25

**Authors:** Audrey Butty, Nolwenn Bühler, Jérôme Pasquier, Julien Dupraz, Vincent Faivre, Sandrine Estoppey, Cloé Rawlinson, Semira Gonseth Nusslé, Murielle Bochud, Valérie D’Acremont

**Affiliations:** ^1^ Department of Epidemiology and Health Systems, Center for Primary Care and Public Health (Unisanté), University of Lausanne, Lausanne, Switzerland; ^2^ STS Lab, Institute of Social Sciences, Faculty of Social and Political Sciences, University of Lausanne, Lausanne, Switzerland; ^3^ Swiss Tropical and Public Health Institute, University of Basel, Basel, Switzerland

**Keywords:** COVID-19, adherence, SARS-CoV-2, preventive measures, population-based sample, representative, preventive behaviours

## Abstract

**Objectives:** We quantified adherence to COVID-19 preventive measures and explored associated factors, after the first and during the second Swiss epidemic waves.

**Methods:** With an observational cohort study in a representative sample of individuals aged 15 years and more, we analysed the association between self-reported adherence to COVID-19 preventive measures (respect of simple hygiene rules; respect of social distancing rules; wearing a mask) and socio-demographic factors, the existence of a chronic disease, and the existence of a previous confirmed COVID-19 episode.

**Results:** Highest adherence was to simple hygiene rules, followed by social distancing rules and mask wearing, with a slight decrease for simple hygiene rules and a strong increase for mask wearing between visits. Men were significantly less likely to respect simple hygiene rules and wear a mask in public. Participants aged 65 years and more (versus 25–64 years) and those with at least one chronic disease (versus none) were two times more likely to respect social distancing rules and wear a mask.

**Conclusion:** Adherence to social distancing rules and mask wearing was rather poor, especially compared to other countries.

## Introduction

From the beginning of the Coronavirus Disease 2019 (COVID-19) pandemic, public health authorities have widely recommended several specific preventive measures, such as washing hands, social distancing and mask wearing. These interventions have proven to be partly effective in reducing the transmission of common respiratory viruses [1–3], including severe acute respiratory syndrome coronavirus 2 (SARS-CoV-2) [2, 4]. Adherence to preventive measures by the population is important to reduce the spread and burden of COVID-19. Studies on preventive behaviours in different geographical areas and age groups of the population are crucial to understand social and cultural factors associated with non-adherence to target public health interventions in the population during the course of the pandemic, as well as to help preparing future outbreaks.

Studies were conducted in several countries to assess adherence to COVID-19 preventive measures [5–8]. However, these studies did not use a representative sample of the population. Few population-based studies were conducted on COVID-19 preventive behaviours so far [9–15]. One nationally representative survey administered to 5009 adults in the United States looked at the difference in COVID-19 preventive behaviours between rural and urban areas, finding that rural residents were less likely to follow preventive health measures [10]. Another population-based study, conducted in Hong Kong, looked at the association between social capital and preventive behaviours during the COVID-19 pandemic and reported that lack of perceived sense of community belonging was associated with decreased odds of preventive behaviours [9]. In Switzerland, a population-based online survey of individuals aged between 15 and 79 years was conducted to monitor and analyse preventive behaviours (social distancing, mask wearing, vaccination and the use of the SwissCovid app), and especially to understand how perceived social norms of preventive behaviours evolved during the COVID-19 pandemic [16]. However, no data was collected on simple hygiene rules and around the first epidemic wave. Another study analysed adherence to COVID-19 hygiene and social distancing rules among adolescents during the first epidemic wave in Switzerland [17]. There is a lack of studies assessing adherence to COVID-19 preventive measures at different time-points, allowing monitoring changes in adherence between the beginning of the pandemic and the subsequent epidemic waves.

We carried out an observational cohort study in a representative sample of adolescents and adults in the Canton of Vaud, Switzerland. The aim of this analysis was to quantify self-reported adherence to individual COVID-19 preventive measures, namely the respect of simple hygiene and social distancing rules, as well as wearing a mask in public, within the specific setting of the Canton of Vaud, and explore factors associated with adherence, after the first and during the second Swiss epidemic waves.

## Methods

### Study Population and Design

The present study is part of a seroepidemiological repeated cross-sectional study of SARS-CoV-2 infection (SérocoViD) conducted in the Canton of Vaud (French-speaking region of Switzerland, 806,088 inhabitants on December 31, 2019). Participants of the first survey aged 15 years and more were followed up for a second survey. Here, we present the study design and the results of this cohort of individuals aged 15 years and more from the first two surveys of SérocoViD. The baseline (first survey) and follow-up (second survey) visits took place, respectively, after the first (3 May and 7 July 2020) and during the second Swiss epidemic wave (20 October and 12 December 2020).

The SérocoViD study is part of the nationwide research program, called «Corona Immunitas» of the Swiss School of Public Health (SSPH+), which is aimed at determining the development of SARS-CoV-2 immunity in Switzerland. The program delivers epidemiological data to support health authorities in deciding about the appropriate and effective measures to protect the population and to try mitigating the magnitude of further waves of infection, in order to avoid overloading the Swiss health care system (www.corona-immunitas.ch I www.ssphplus.ch) [18].

For the baseline visit, participants were selected from the official population registries by the Federal Office of Statistics, using a Poisson sampling, while stratifying by age groups: 15–19 years, 20–39 years, 40–64 years, 65–74 years, 75 years and more. Exclusion criteria included institutionalized individuals, individuals without their capacity of giving consent, diplomats and asylum seekers. We obtained written informed consent from all participants. The Ethics committee of the Canton of Vaud reviewed and approved the study protocol (2020-00887) on 24 April 2020. We invited all participants of the baseline survey to a follow-up visit.

### Recruitment Procedures

Initially, sampled participants were contacted by postal mail (with, respectively for the baseline and follow-up visit, up to three and two reminders by letter). In addition, for the baseline visit, whenever a phone number was available (i.e., in ∼60% of the cases) participants were called by study staff. In the invitation letter, we asked participants to register online on the study website and to complete the study questionnaires. Then, participants could arrange a study visit at a chosen date and time, in one of the four study sites. For the follow-up visit, there was only one study site. In order to prevent a possible participation bias from participants with a lower digital literacy, all participants had the option to complete the study questionnaires during the visit with the help of a study nurse. In addition, and upon request, we offered a home visit to participants defined on medical grounds as particularly vulnerable to COVID-19.

### Data and Biological Material Collection

Questionnaires included the following topics: age, gender, current occupation, profession, working sector, change of working conditions, and school year/type of study for children and adolescents; place of residence and people living in the same household; comorbidities, medication, height/weight, pregnancy; COVID-19 specific information: specific symptoms, hospitalizations, testing, isolation and quarantine, suspected and confirmed family members/close persons COVID-19 cases; risk behaviours: following hygiene rules and physical distancing, meeting other people, utilization of masks, self-isolation, self-quarantine of household contact. We built questionnaires using REDCap [19]. For the follow-up visit, questionnaires slightly differed from the baseline visit, to ensure interoperability with the Corona Immunitas national project. Differences relevant for the analysis are explained in Supplementary Text S1.

In order to determine the serological status of the participants, whole blood was collected from venepuncture. In case of a refusal or failure of the venepuncture in individuals aged 14–18 years at baseline, capillary blood from finger prick was collected as an alternative. Whole blood was stored at room temperature and centrifuged within 1 hour from collection. Serum was stored at −20°C for a maximum of 7 days and then at −80°C until serology was performed. We measured anti-SARS-CoV-2 immunoglobulin G (IgG) and A (IgA) antibodies targeting the spike (S) protein in its native trimeric form using a Luminex immunoassay, which was developed by the Lausanne University Hospital (CHUV) in collaboration with the École Polytechnique Fédérale de Lausanne (EPFL) [20]. In the present study, we defined a positive serological result as either IgG and/or IgA positive result.

### Preventive Measures

The main outcome measure was the level of adherence to COVID-19 preventive measures, which included: 1) respect of simple hygiene rules, 2) respect of social distancing rules, and 3) wearing a mask in public. Each of these were coded into a binary dependent variable. We analysed the association between self-reported adherence to these preventive measures and socio-demographic factors (age, gender, education), the existence of a chronic disease, and the existence of a previous confirmed COVID-19 episode.

### Covariates

We included the following covariates in the multivariable analyses: age (15–24 years; 25–64 years; 65 years and more), gender (man; woman; other), education level (no education, lower secondary or less; upper secondary; tertiary), and chronic disease (none; one or more).

### Statistical Analyses

We used a McNemar test to assess the difference in adherence to preventive measures between baseline and follow-up visits. We conducted, for each visit, bivariate analysis to measure the association between categorical independent variables and the three binary preventive measures using weighted Chi-square tests. We performed, for each visit, weighted multivariable logistic regression models to measure the strength of the association between independent variables of interest (gender, age at baseline, education, chronic disease) and each of the three binary preventive measures, taken one-at-a-time, as the dependent variable of interest. We calculated adjusted odds radio (aOR), 95% confidence interval (CI) and *p*-values for each independent variable. Statistical analyses were performed with R [21]. The significance level was set at 0.05.

## Results

### Characteristics of Participants

**FIGURE 1:**
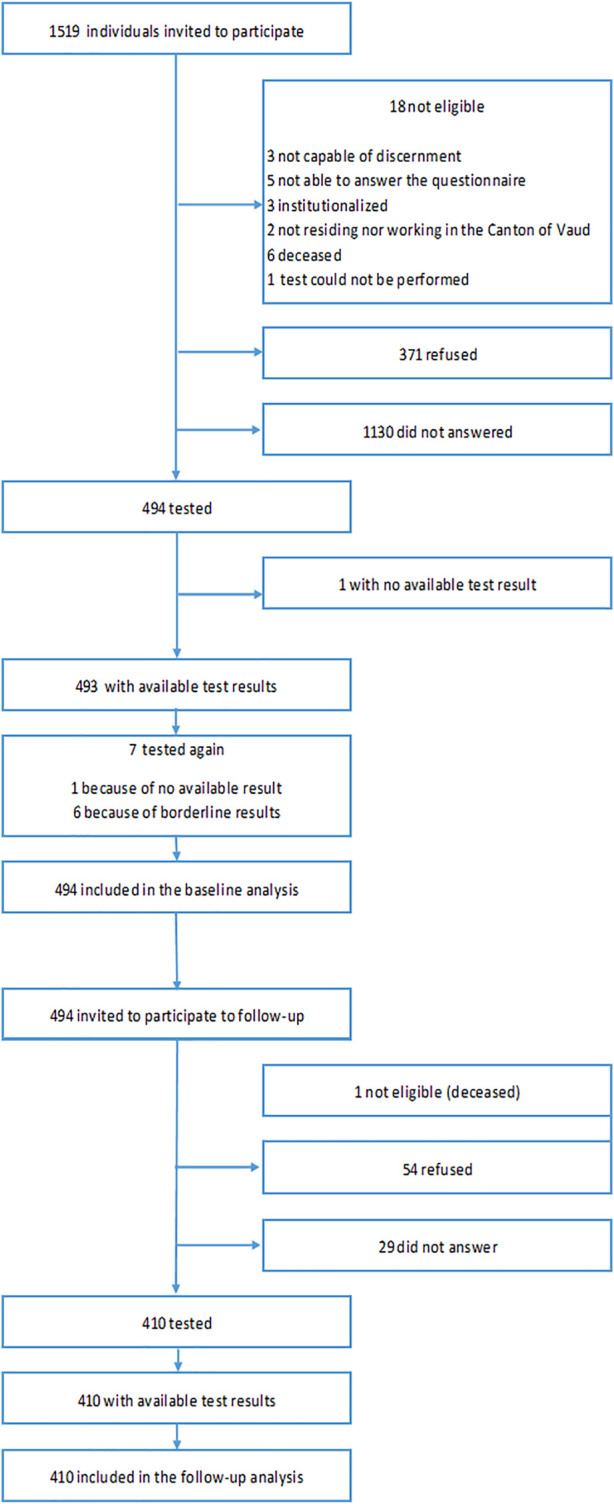
Participation flow chart (SérocoViD study, Vaud, Switzerland, 2020).

Of 1501 individuals eligible to participate initially (Figure 1), 494 took part in the baseline visit (32.9%) between 3 May and 7 July 2020. Of 493 individuals eligible for the follow-up visit, 410 participated (83.2%) between 20 October 2020 and 12 December 2020, and had thus both visits (Supplementary Figure S1).

At baseline, most of the 494 participants were women (54.5%) and aged between 25–64 years (68.6%) (Table 1). Ninety-five participants (11.4%) were aged between 15–24 years and 178 (20.0%) were aged 65 years or older. The proportion of participants with lower secondary education or less was 8.5%, whereas it was higher in higher education levels (42% with upper secondary education and 49.6% with tertiary education). There were 171 (31.3%) participants who reported having at least one chronic disease, 109 (24.6%) who were current smokers, and 207 (44.2%) who were overweight or obese. One (0.1%) participant had experienced at least one confirmed COVID-19 episode. The prevalence of self-reported adherence to preventive measures was 79.7% for always respecting simple hygiene rules, 60% for always respecting social distancing rules, and only 13.3% for always wearing a mask in public. Forty-seven (10.5%) had a positive serological result.

**TABLE 1:** Participants’ characteristics at baseline and follow-up (SérocoViD study, Vaud, Switzerland, 2020).

	Baseline	Follow-up
Number of participants	494 (100.0)	410 (100.0)
Gender		
Women	266 (54.5)	220 (54.1)
Men	228 (45.5)	190 (45.9)
Others	None	None
Age at baseline		
15–24 years	95 (11.4)	77 (11.3)
25–64 years	221 (68.6)	187 (68.7)
65+ years	178 (20.0)	146 (20.0)
Education^a^		
Lower secondary or less	60 (8.5)	76 (10.0)
Upper secondary	235 (42.0)	151 (35.7)
Tertiary	197 (49.6)	182 (54.4)
Smoking status		
Non smokers	277 (55.6)	252 (60.7)
Ex-smokers	107 (19.8)	87 (20.7)
Current smokers	109 (24.6)	71 (18.7)
Weight status		
Normal or underweight	283 (55.8)	230 (54.8)
Overweight or obese	207 (44.2)	178 (45.2)
Chronic disease^b^		
None	308 (68.7)	289 (74.8)
One or more	171 (31.3)	121 (25.2)
Previous confirmed COVID-19 episode^c^		
None	493 (99.9)	350 (82.8)
One or more	1 (0.1)	60 (17.2)
Respect of simple hygiene rules		
Yes, always^d^	392 (79.7)	301 (73.0)
Not always^e^	96 (20.3)	109 (27.0)
Respect of social distancing rules		
Yes, always^d^	306 (60.0)	237 (60.1)
Not always^e^	183 (40.0)	172 (39.9)
Wearing a mask in public		
Yes, always^f^	81 (13.3)	238 (55.5)
Not always^g^	409 (86.7)	172 (44.5)
Serological test		
Positive	47 (10.5)	57 (16.9)
Negative	447 (89.5)	353 (83.1)

a Baseline: For adults (>20 years), highest level of education. For adolescents (15–20 years), current education. Follow-up: For adults (>20 years) and adolescents (15–20 years), highest level of education.

b Baseline: For adults, presence of hypertension, diabetes, cardiovascular disease, respiratory disease, immunity deficiency, cancer, or other chronic disease. For adolescents, presence of a non-specified chronic disease. Follow-up: For adults and adolescents, presence of hypertension, diabetes, cardiovascular disease, respiratory disease, immunity deficiency, cancer, or other chronic disease.

c Baseline: Presence of a previous positive PCR test result. Follow-up: Presence of a previous positive PCR or rapid antigen or baseline serological test result.

d Baseline: “Yes.” Follow-up: “Always”.

e Baseline: “Mostly yes,” “Mostly no,” or “No.” Follow-up: “Frequently,” “Occasionally,” “Very rare,” or “Never.”

f Baseline: “Yes, always.” Follow-up: “Always.”

g Baseline: “Yes, sometimes,” or “No.” Follow-up: “Frequently,” “Occasionally,” “Very rare,” or “Never.”

Presented as number with percentage.

Participants’ characteristics and reported adherence to preventive measures were similar between both visits, except for the measure of always wearing a mask in public, which was more frequently respected at follow-up (13.3% at baseline versus 55.5% at follow-up). At follow-up, 60 (17.2%) participants had experienced at least one confirmed COVID-19 episode and 57 (16.9%) had a positive serological result.

We report the principal baseline characteristics of participants and non-participants to the follow-up visit in Supplementary Table S1. There was no difference in participation according to characteristics, except for weight status.

### Change in Adherence to Preventive Measures Over Time

Out of 405 participants, 325 (79.9%) reported always respecting simple hygiene rules at baseline and 299 (73.5%) at follow-up (*p* = 0.011). Out of 406 individuals, 63 (13.0%) reported always wearing a mask in public at baseline, compared to 235 (55.4%) at follow-up (*p* < 0.001). The proportion of participants who declared always respecting social distancing rules was similar at baseline (60.8%) and follow-up (60.3%).

### Risk Factors for Adherence to Preventive Measures

**FIGURE 2:**
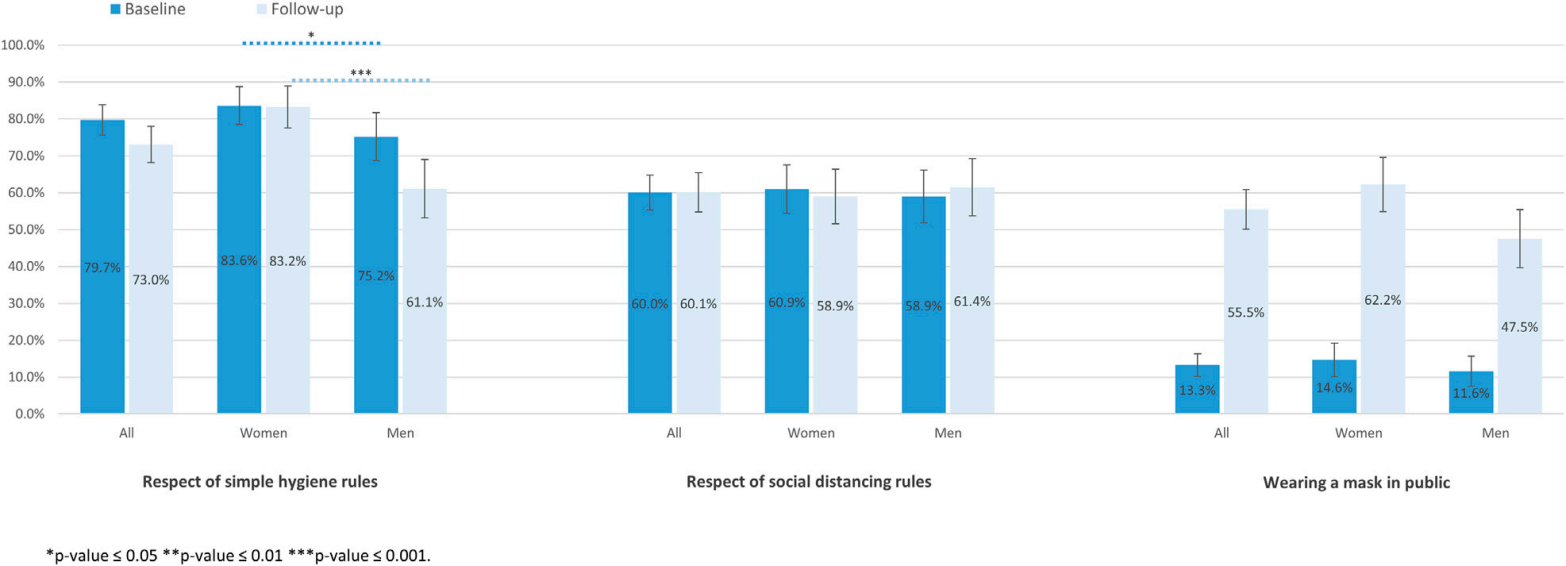
Adherence to preventive measures according to gender at baseline and follow-up (SérocoViD study, Vaud, Switzerland, 2020).

Adherence to preventive measures according to gender at baseline and follow-up are shown in Figure 2. The proportion of women who reported always respecting simple hygiene rules at baseline was significantly higher than that of men (83.6% versus 75.2%, *p* = 0.045). The difference was even more significant at follow-up (83.2% versus 61.1%, *p* < 0.001). Men and women reported similar adherence to social distancing rules or mask wearing in public at baseline and at follow-up.

**FIGURE 3:**
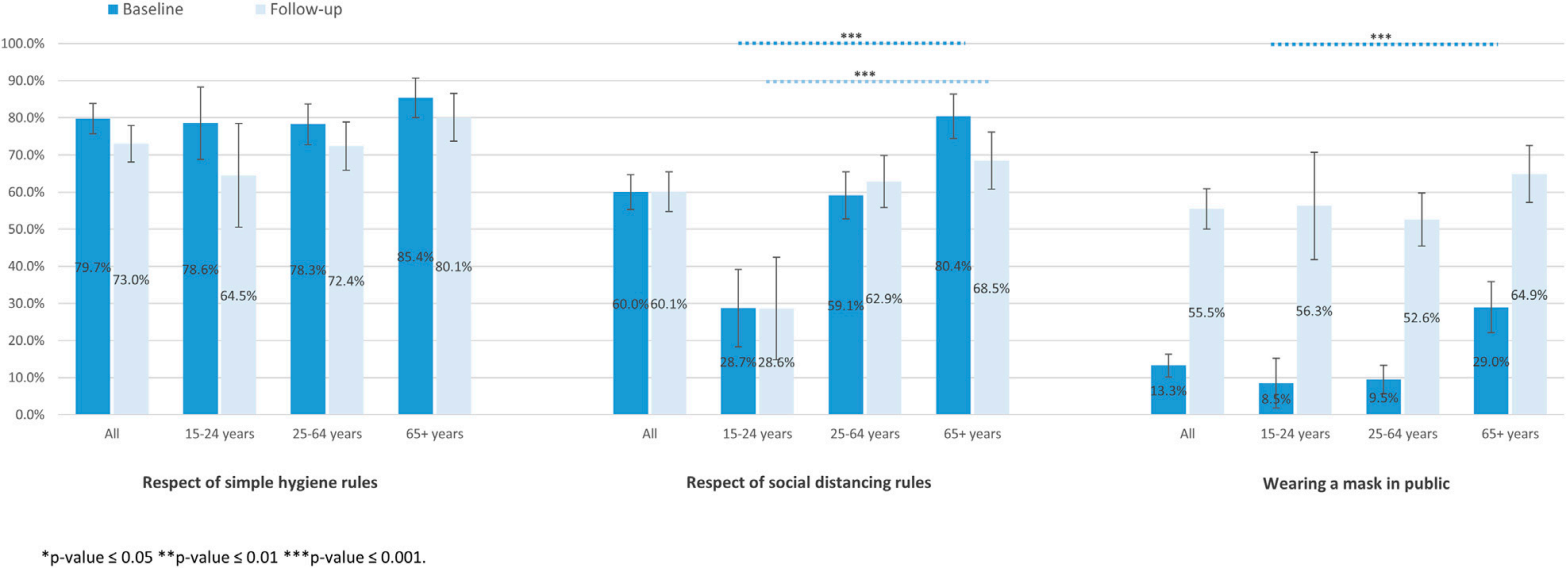
Adherence to preventive measures according to age at baseline and follow-up (SérocoViD study, Vaud, Switzerland, 2020).

The proportion of participants who respected social distancing rules was significantly higher in older age groups, at both baseline and follow-up (Figure 3). There was no significant association between age groups and the respect of simple hygiene rules. Older participants more frequently reported to always wear a mask in public at baseline compared to younger individuals (29.0%, 9.5%, and 8.5%, *p* < 0.001, for 65 years and more, 25–64 years and 15–24 years, respectively).

**FIGURE 4:**
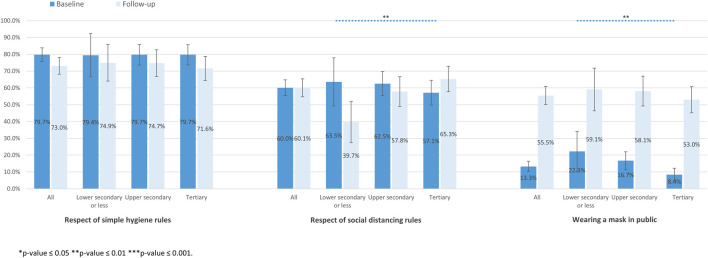
Adherence to preventive measures according to education level at baseline and follow-up (SérocoViD study, Vaud, Switzerland, 2020).

We found no association of education level with the respect of simple hygiene rules, at both baseline and follow-up (Figure 4). At follow-up, the respect of social distancing rules was significantly higher among participants with higher education levels than among other education categories. Participants with lower education levels reported to more frequently wear a mask in public at baseline (22.3%, 95% CI 10.4–34.1) compared to those with upper secondary (16.7%, 95% CI 11.4–22.0) and tertiary education level (8.4%, 95% CI 4.6–12.2).

Compared to participants with no chronic disease, the proportion of participants with at least one chronic disease who respected simple hygiene rules tended to be higher at baseline and was significantly higher at follow-up. The proportion of participants with at least one chronic disease who respected social distancing rules was also significantly higher compared to those without, both at baseline and follow-up. This was also true for wearing a mask in public (Supplementary Figure S2).

We did not include the variable “previous confirmed episode of COVID-19” as a covariate in the multivariable analysis because only one person met this criteria at baseline (Supplementary Figure S3).

In multivariable analyses including gender, age, education, and chronic disease as covariates (Table 2), men were less likely to respect simple hygiene rules compared to women, both at baseline and follow-up (aOR 0.56, 95% CI 0.33–0.96 and aOR 0.30, 95% CI 0.17–0.51, respectively). Men were less likely to wear a mask in public compared to women at both baseline and follow-up, but this was only statistically significant at follow-up (aOR 0.55, 95% CI 0.35–0.87). Participants aged 15–24 years were significantly less likely to respect social distancing compared to those aged 25–64 years at both visits (aOR 0.29, 95% CI 0.16–0.55 and aOR 0.33, 95% CI 0.14–0.79, respectively). At baseline, participants aged 65 years and more were two times more likely to respect social distancing rules and to wear a mask, compared to those aged 25–64 years. Participants reporting at least one chronic disease were approximatively two times more likely to respect social distancing rules (at follow-up) and wear a mask (at both visits) compared to those having no chronic disease. Education level did not show an association with any of the preventive measures.

**TABLE 2:** Multivariable analysis of participants’ adherence to preventive measures at baseline and follow-up (SérocoViD study, Vaud, Switzerland, 2020).

	Respect of simple hygiene rules		Respect of social distancing rules		Wearing a mask	
	Baseline (N = 474)	Follow-up (N = 409)	Baseline (N = 475)	Follow-up (N = 408)	Baseline (N = 473)	Follow-up (N = 409)
Covariates	aOR (95% CI)	aOR (95% CI)	aOR (95% CI)	aOR (95% CI)	aOR (95% CI)	aOR (95% CI)
Gender						
Women	reference	reference	reference	reference	reference	reference
Men	0.56 (0.33; 0.96)*	0.30 (0.17; 0.51)***	0.82 (0.52; 1.29)	1.03 (0.64; 1.67)	0.73 (0.40; 1.33)	0.55 (0.35; 0.87)**
Age at baseline						
15–24 years	1.04 (0.51; 2.09)	0.51 (0.22; 1.23)	0.29 (0.16; 0.55)***	0.33 (0.14; 0.79)**	1.03 (0.35; 3.03)	1.14 (0.52; 2.51)
25–64 years	reference	reference	reference	reference	reference	reference
65+ years	1.66 (0.89; 3.08)	1.31 (0.72; 2.42)	2.39 (1.42; 4.00)***	1.17 (0.70; 1.96)	2.63 (1.40; 4.94)**	1.34 (0.82; 2.19)
Education^a^						
Lower secondary or less	reference	reference	reference	reference	reference	reference
Upper secondary	1.05 (0.42; 2.65)	0.73 (0.31; 1.71)	0.93 (0.43; 2.02)	1.47 (0.68; 3.15)	0.72 (0.32; 1.64)	1.00 (0.48; 2.11)
Tertiary	1.09 (0.41; 2.90)	0.67 (0.28; 1.58)	0.76 (0.34; 1.68)	2.05 (0.95; 4.44)	0.50 (0.20; 1.25)	0.89 (0.42; 1.87)
Chronic disease^b^						
None	reference	reference	reference	reference	reference	reference
One or more	1.13 (0.62; 2.06)	1.56 (0.76; 3.19)	1.51 (0.88; 2.59)	1.93 (1.09; 3.43)*	2.36 (1.22; 4.55)**	1.90 (1.08; 3.32)*

a Baseline: For adults (>20 years), highest level of education. For adolescents (15–20 years), current education. Follow-up: For adults (>20 years) and adolescents (15–20 years), highest level of education.

b Baseline: For adults, presence of hypertension, diabetes, cardiovascular disease, respiratory disease, immunity deficiency, cancer, or other chronic disease. For adolescents, presence of a non-specified chronic disease. Follow-up: For adults and adolescents, presence of hypertension, diabetes, cardiovascular disease, respiratory disease, immunity deficiency, cancer, or other chronic disease.

*p-value ≤ 0.05 **p-value ≤ 0.01 ***p-value ≤ 0.001.

Adjusted odds ratios come from a multivariable logistic regression analysis including gender, age, education and chronic disease as covariables. aOR, adjusted odds ratio. N, number of participants. CI, confidence interval.

## Discussion

The present study provides insights into adherence to COVID-19 preventive measures in Switzerland at the very beginning of the pandemic. In this population-based sample, we found that adherence to the main preventive measures recommended by public health authorities was highest for simple hygiene rules, followed by social distancing rules and mask wearing, with a slight decrease for simple hygiene rules and a strong increase for mask wearing, during the second epidemic wave compared to right after the first wave of the COVID-19 pandemic.

Adherence to simple hygiene practices was even higher in other countries than in our survey, with 86% in the United Kingdom [13], 98% in Saudi Arabia [11], approximately 89% in the United States (with no difference between rural and urban areas) [10, 14], 90% in Spain [15], and more than 92% in Hong Kong [9]. High level of adherence in some of these countries could be explained by the previous epidemics of MERS in Saudi Arabia [22] and SARS in Hong Kong [23], as suggested by Alkahldi and al [11]. In Hungary, 89% of the population declared washing hands when arriving at home and 69% while outside home [12]. Preventive measures considered as simple hygiene practices were similar between these studies, except for the one conducted in Saudi Arabia, in which mask wearing was included in the category of simple hygiene practices. All of these studies used a representative sample of the adult population aged 18 years and more. The study in Hong Kong also included adolescent participants aged 15 years and more, which makes it more similar to our study. However, apart from differences in characteristics of the study populations, social, cultural, economic and historic factors may also explain differences in adherence to preventive measures.

In our study, only approximately 60% of the population declared respecting social distancing, regardless of the time period. This is consistent with another population-based Swiss survey in individuals aged between 15 and 79 years, which has been monitoring adherence to some preventive measures weekly since September 2020, right before the second Swiss epidemic wave [16]. The proportion of the population reporting practicing social distancing varied across different countries with 45% in the United Kingdom [13], 40% in Hungary [12], 85% in Spain [15], approximately 89% in the United States (with no difference between rural and urban areas) [10], and 98% in Saudi Arabia [11]. These studies all used a representative sample of the adult population and social distancing categories included similar types of measures.

The proportion of the population who reported wearing a mask in public increased significantly between the two visits of our study, which can be explained by mask wearing being mandatory in a range of indoor spaces during the second epidemic wave in Switzerland, whereas it was not during the first epidemic wave. However, despite mask wearing being mandatory, only half of the population strictly adhered to this public health measure in the middle of the second epidemic wave. This is lower than what was reported by Friemel and al. in the Swiss population during the same period of our follow-up survey [16]. In Saudi Arabia, a similar proportion of the population declared wearing a mask (56%) between April and June 2020, but with no information on mask wearing recommendation at this time [11]. In addition, adherence to face masks was higher in the United States between May and June 2020, with a significant difference between urban residents (85%) and rural residents (74%) [10]. Difference between urban and rural areas can be due to risk perception being higher in crowded urban areas [24]. Face mask adherence was of 92% in Spain [15], and even higher in Hong Kong (more than 97%) between February and April 2020 [9]. However, only 20% of the Hungarian population reported to wear a mask between March and April 2020, despite good adherence to all other public health recommendations [12]. These strong differences in mask wearing across populations can likely be explained by differences in public health recommendations, and in accessibility to masks across countries, as well as by social, cultural, economic and historic influences on the meaning of mask wearing [25–28]. Indeed, before the pandemic, in most European and other Western countries, mask wearing was not a common behaviour, whereas in Asian countries, face masks were already regularly worn because of previous outbreaks and air pollution [29]. The social meaning of face masks can be influenced by actions from governments and political leaders. Messages from governments can stigmatize mask wearing (such as when associating it with the sick), or on the contrary, promote it (such as when introducing mask mandates) [30]. Mask mandates are associated with increased adherence to mask-wearing [24].

Adherence to mask wearing is often lower compared to the other main preventive measures, which could be due to negative perceptions associated with the use of face masks. Especially, in men, face masks are seen as infringing upon their independence, and in women, as uncomfortable [31].

We confirm prior studies showing that men have worse overall adherence to preventive measures than women [32, 33], including during the COVID-19 pandemic [7, 10, 12, 17, 34]. Men are less likely to practice simple hygiene rules [35, 36] and social distancing [11, 37], and to wear a mask [24, 38–40] than women. It was reported that “risk perception and health beliefs (especially perceived severity of COVID-19 related conditions)” can explain the difference in adherence to preventive measures between men and women, and that poor adherence to preventive measures and poor risk perception “may contribute to the lower life expectancies in general and the higher mortality rate due to COVID-related complications among males” [41]. It was shown that women have higher perceived risk and fear of SARS-CoV-2 infection than men, explaining why they tend to engage more in preventive behaviours [42]. Further research is needed to better understand social, relational and especially gender factors associated with non-adherence to preventive measures in men to reduce their risk of SARS-CoV-2 infection.

Adherence to preventive measures was stronger for older participants and for those having at least one chronic disease, which may be explained by both being risk factors of severe COVID-19. Older age is a strong predictor of good adherence to COVID-19 preventive measures, as reported by several studies [12, 41]. Older people are more likely to wear a mask [24] and to practice social distancing [13] than younger people. Friemel and al. also reported that adherence to face mask, social distancing, vaccination and testing in the Swiss population was higher at older age [43]. Hills and al. also reported that vulnerability to severe COVID-19 was positively associated with adherence to social distancing rules [44]. It was also shown that cancer survivors were more likely to adopt preventive measures, such as social distancing and mask wearing [45]. By contrast, a population-based study in Saudi Arabia reported lower odds of adherence to hygiene and social distancing rules in people aged 65 years and more compared to those aged between 18 and 24 years [11]. In this study, older individuals had lower risk perception (possibly related to some optimism bias [46]—the belief that the risk is low—and to religious beliefs having a stronger influence on health behaviours and perceptions in elderly people in Saudi Arabia [47]), which might explain this difference.

We found no association of self-reported adherence to preventive measures with education level. It may be that participation rate differed across education level thereby obscuring a potential true underlying association or that the overall high level of education in Switzerland compared to other countries did not allow to detect a difference in the few participants with a lower education. Several studies have indeed reported that poor adherence to COVID-19 preventive measures was associated with lower education level [5, 10]. Regarding other socio-economics factors, a positive association between lack of perceived social harmony and wearing masks was shown in people with income loss (compared to those with a gain or no change in income) [9]. However, a representative survey administered to 5009 adults in the United States found that higher income was associated with increased adherence to several preventive behaviours [10]. By contrast, a cohort study among young adults in Switzerland reported that non-adherence was higher in those with higher education or higher socio-economic status [17]. Findings about the association between education level and adherence to preventive measures are for the least contradictory.

A strength of this study is the population-based sample. Our study has several limitations, such as an overall limited number of participants. All of those studies mentioned above had larger sample sizes than our study (approximately 990–5200 participants). The low participation rate at baseline (32.9%) may affect the external validity of the results. In addition, the results may be biased due to the fact that some people could have been more likely to participate if they suspected to have been infected with SARS-CoV-2 but never had a confirmation by antigen rapid test or polymerase chain reaction (PCR). Finally, the questionnaires were slightly different between the two visits, due to time and logistic constraints.

Our study shows that adherence to simple hygiene rules was high among all groups, whereas it was relatively low regarding social distancing rules and mask wearing. Nonetheless, all three main preventive behaviours are known to be, at least partly, effective in reducing the transmission of SARS-CoV-2 [2, 4], and it reassuring to see that people at risk, older people or those with at least one chronic disease, have better adherence to social distancing rules and mask wearing than the rest of the population. Yet, public health interventions are still crucial to increase adherence in men, young individuals and those with lower education level. Community adherence to preventive measures is essential to reduce transmission of SARS-CoV-2 and protect at risk individuals.

Socio-demographics characteristics are associated with adherence, however they only partly explain adherence to preventive measures. A comprehensive review identified and summarized factors of adherence to COVID-19 preventive measures [48]. These include: “individual socio-demographic and behavioural factors, living and working conditions, COVID-19 knowledge, attitudes and risk perception, exposure to sources and information level, leisure activities, social support, trust, social norms, psychosocial well-being, socio-economic position, and the socio-economic and political context.”(48) Adherence to COVID-19 preventive measures can be increased if we understand better the perception of preventive measures and the challenges of appropriating them in daily life. Studies have shown that adherence to COVID-19 preventive measures is increased if people have adequate knowledge of how SARS-CoV-2 infection spreads, agree with mandatory mask use and perceive the situation as being at high risk for themselves or others [15], as well as believe in the effectiveness of preventive measures [49]. Several studies using the Health Belief Model also reported that factors such as perceived benefits, perceived susceptibility or perceived severity are positively associated with COVID-19 preventive behaviours, whereas perceived barriers are negatively associated with such behaviours [50, 51]. In addition, Friemel and al. reported that the use of news media as communication was associated with behaviour-related perceptions of efficacy and norms, as well as disease-related perceptions of threat; these perceptions were in turn positively associated with adherence to social distancing rules [52]. Trust in official government and social networks are also important drivers of adherence to preventive measures [53]. Therefore, better attention should be paid to individual knowledge and perceptions, as well as social, cultural and psychological factors associated with adherence and non-adherence. Involvement of communities is essential for understanding those factors; however, communities are often poorly involved in the planning and implementation of public health interventions. Community engagement approaches have proven to be effective for prevention and control of past epidemics [54]. Similarly, they should be used when managing the COVID-19 pandemic to support the implementation of preventive measures. By designing appropriate interventions, building trust and community entry, communicating risk [54], increasing public risk perceptions, knowledge and education, as well as addressing doubts and debates [55], community engagement approaches may lead to increased adherence to preventive measures. Community engagement is also essential to increase vaccine uptake [56]. People with better adherence to preventive measures are also more likely to accept vaccination [6]. In the context of the SérocoViD study, a qualitative part (SociocoViD) was developed to explore these aspects in more depth. It aimed at complementing our quantitative results, by exploring in-depth experiences of risk and protection during the pandemic and shedding light on how living conditions shaped those. It will provide further information on social, cultural and psychological factors associated with adherence.

This study offers one of the first findings on the adherence to COVID-19 preventive measures in a population-based sample in Switzerland. Despite high adherence to simple hygiene rules, adherence to social distancing rules and mask wearing was rather poor, especially compared to other countries, despite the communication strategy on public health recommendations put in place by health authorities. However, it is reassuring that high-risk individuals were strongly engaged in all three preventive measures. Qualitative research is needed to understand more finely meanings, values and norms underlying preventive behaviours. Community engagement might be used to discuss the challenges of adhering to preventive measures for concerned populations, especially men, young individuals and those with lower education level.

## Supplementary Material

Supplementary Figure S1 Participation rates (SérocoViD study, Vaud, Switzerland, 2020).

Supplementary Figure S2 Adherence to preventive measures according to the existence of chronic disease at baseline and at follow-up (SérocoViD study, Vaud, Switzerland, 2020).

Supplementary Figure S3 Adherence to preventive measures according to previous confirmed COVID-19 episode at baseline and follow-up (SérocoViD study, Vaud, Switzerland, 2020).
