# Supplementary material for: Adherence to Coronavirus Disease 2019 Preventive Measures in a Representative Sample of the Population of the Canton of Vaud, Switzerland
Source: Int J Public Health. 2022 Aug 25;67:1605048. doi: 10.3389/ijph.2022.1605048 (PMC9453818; doi:10.3389/ijph.2022.1605048)
Supplement: Supplementary file 1 [file Table1.pdf]

Supplementary Table S1. Comparison of the characteristics of participants and non-participants to the follow-up visit (SérocoViD study, Vaud, Switzerland, 2020).

	All	Did not participate	Participated	p-value
Number of participants	494 (100.0)	84 (16.8)	410 (83.2)	
Gender				
Women	266 (54.5)	46 (58.6)	220 (53.6)	0.465
Men	228 (45.5)	38 (41.4)	190 (46.4)	
Others	none	none	none	
Age at baseline				
15–24 years	95 (11.4)	18 (14.6)	77 (10.8)	0.535
25–64 years	221 (68.6)	34 (64.6)	187 (69.4)	
65+ years	178 (20.0)	32 (20.7)	146 (19.8)	
Education[a]				
Lower secondary or less	60 (8.5)	12 (11.1)	48 (7.9)	0.061
Upper secondary	235 (42.0)	49 (52.4)	186 (39.9)	
Tertiary	197 (49.6)	23 (36.5)	174 (52.2)	
Smoking status				
Non smokers	277 (55.6)	44 (48.4)	233 (57.1)	0.168
Ex smokers	107 (19.8)	19 (18.0)	88 (20.2)	
Current smokers	109 (24.6)	21 (33.6)	88 (22.7)	
Weight status				
Normal or underweight	283 (55.8)	55 (68.1)	228 (53.3)	**0.029**
Overweight or obese	207 (44.2)	28 (31.9)	179 (46.7)	
Chronic disease[b]				
None	308 (68.7)	51 (67.4)	257 (69.0)	0.795
One or more	171 (31.3)	32 (32.6)	139 (31.0)	
Previous confirmed COVID-19 episode[c]				
None	439 (99.1)	84 (100.0)	409 (99.8)	0.655
One or more	1 (0.1)	0 (0.0)	1 (0.2)	
Respect of simple hygiene rules				
Yes, always[c]	392 (79.7)	67 (78.5)	325 (80.0)	0.798
Not always[e]	96 (20.3)	16 (21.5)	80 (20.0)	
Respect of social distancing rules				
Yes, always[d]	306 (60.0)	50 (51.6)	256 (61.7)	0.131
Not always[e]	183 (40.0)	33 (48.4)	150 (38.3)	
Wearing a mask in public				
Yes, always[f]	81 (13.3)	18 (15.0)	63 (12.9)	0.610
Not always	409 (86.7)	66 (85.0)	343 (87.1)	

[a]For adults (> 20 years), highest level of education. For adolescents (15-20 years), current education.

[b]For adults, presence of hypertension, diabetes, cardiovascular disease, respiratory disease, immunity deficiency, cancer, or other chronic disease. For adolescents, presence of a non-specified chronic disease.

[c]Presence of a previous positive PCR test result.

[d]"Yes"

[e]"Mostly yes", "Mostly no", or "No".

[f]"Yes, sometimes", or "No".
