# Supplementary material for: Adherence to Coronavirus Disease 2019 Preventive Measures in a Representative Sample of the Population of the Canton of Vaud, Switzerland
Source: Int J Public Health. 2022 Aug 25;67:1605048. doi: 10.3389/ijph.2022.1605048 (PMC9453818; doi:10.3389/ijph.2022.1605048)
Supplement: Supplementary file 2 [file Image2.pdf]

Supplementary Figure S2. Adherence to preventive measures according to the existence of chronic disease at baseline and at follow-up (SérocoViD study, Vaud, Switzerland, 2020).

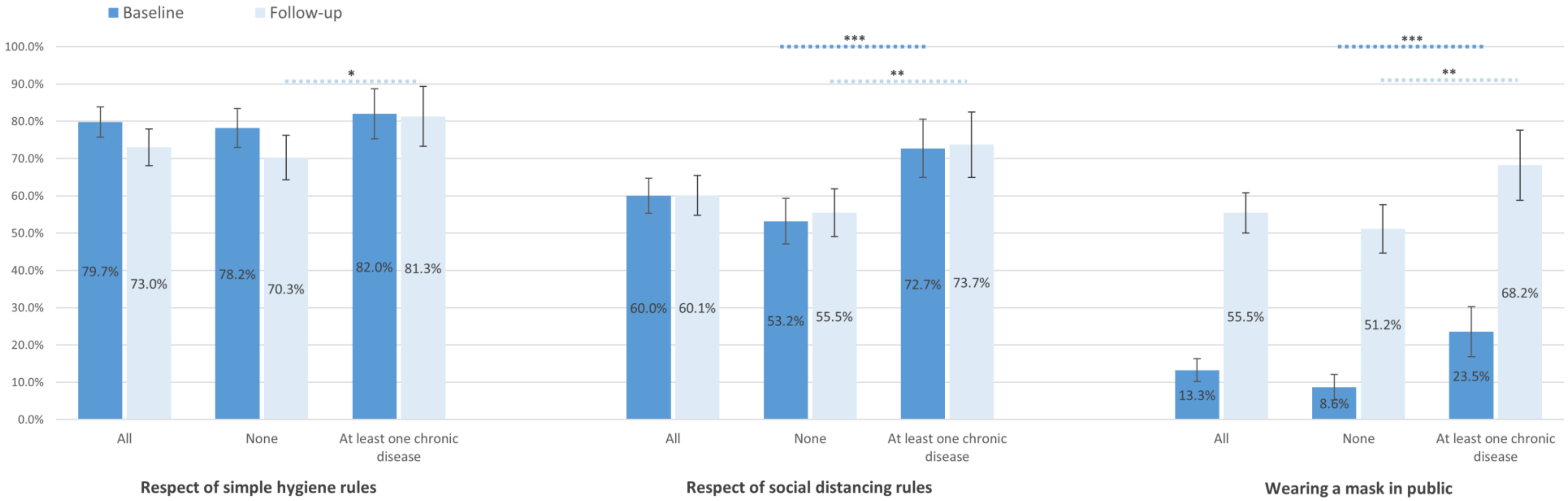

*p-value ≤ 0.05 **p-value ≤ 0.01 ***p-value ≤ 0.001.
