# Supplementary material for: Adherence to Coronavirus Disease 2019 Preventive Measures in a Representative Sample of the Population of the Canton of Vaud, Switzerland
Source: Int J Public Health. 2022 Aug 25;67:1605048. doi: 10.3389/ijph.2022.1605048 (PMC9453818; doi:10.3389/ijph.2022.1605048)
Supplement: Supplementary file 4 [file DataSheet1.pdf]

Supplementary Text S1. Methods for preventive measures and covariates (SérocoViD study, Vaud, Switzerland, 2020).

For both visits, we asked one question for each preventive measure and gave corresponding examples. At the baseline visit, we asked participants if they were following the recommended preventive measures to protect themselves and others form coronavirus infection: (i) “following simple hygiene rules (washing hands regularly, sneezing into the elbow, using a disposable tissue, etc.)”, (ii) “respecting 'social distancing' rules (avoid shaking hands or kissing, staying at home, avoid leaving home unless absolutely necessary, etc.)?”, and (iii) “wearing a mask in public?”. We categorized respect of each preventive measure as “Yes, always” (coded as 1) if the answer was “Yes” for simple hygiene and social distancing rules or if the answer was “Yes always” for wearing a mask in public, and “Not always” (coded as 0) if the answer was either “Mostly yes”, Mostly no” or “No” for simple hygiene and social distancing rules, or if the answer was either “Yes, sometimes” or “No” for wearing a mask in public. At the follow-up visit, we asked participants how many times, in the last seven days, they (i) “implemented the recommended hygiene measures (washing hands regularly, sneezing into the elbow, using tissues, etc.)?”, (ii) “implemented the recommended measures for ‘social distancing’ (no handshaking or hugging, keep the distance of 2 meters, etc.)?, and (iii) “wore a mask to protect yourself and others from the coronavirus (SARS-CoV-2)?”. We categorized respect of each preventive measure as “Yes, always” (coded as 1) if the answer was “Always”, and “Not always” (coded as 0) if the answer was either “Frequently”, “Occasionally”, “Very rare” or “Never”.

At the baseline visit, we asked adults (> 20 years) to report their highest education level and adolescent (15-20 years) their current education level. For the baseline visit, we defined the presence of a chronic disease as either the presence of hypertension, diabetes, cardiovascular disease, respiratory disease, immunity deficiency, cancer, or other chronic disease for adults, and the presence of a non-specified chronic disease for adolescents. For the follow-up visit, the presence of a chronic disease was defined as either the presence of hypertension, diabetes, cardiovascular disease, respiratory disease, immunity deficiency, cancer, or other chronic disease (for both adults and adolescents). We defined a previous confirmed COVID-19 episode, for the baseline visit, as the presence of at least one previous positive PCR test result, and for the follow-up visit, as the presence of at least one previous positive PCR or rapid antigen or baseline serological test result.
