# Supplementary figures and images for: Adherence to Coronavirus Disease 2019 Preventive Measures in a Representative Sample of the Population of the Canton of Vaud, Switzerland
Source: Int J Public Health. 2022 Aug 25;67:1605048. doi: 10.3389/ijph.2022.1605048 (PMC9453818; doi:10.3389/ijph.2022.1605048)

**Supplementary Figure S1. Participation rates (SérocoViD study, Vaud, Switzerland, 2020).**

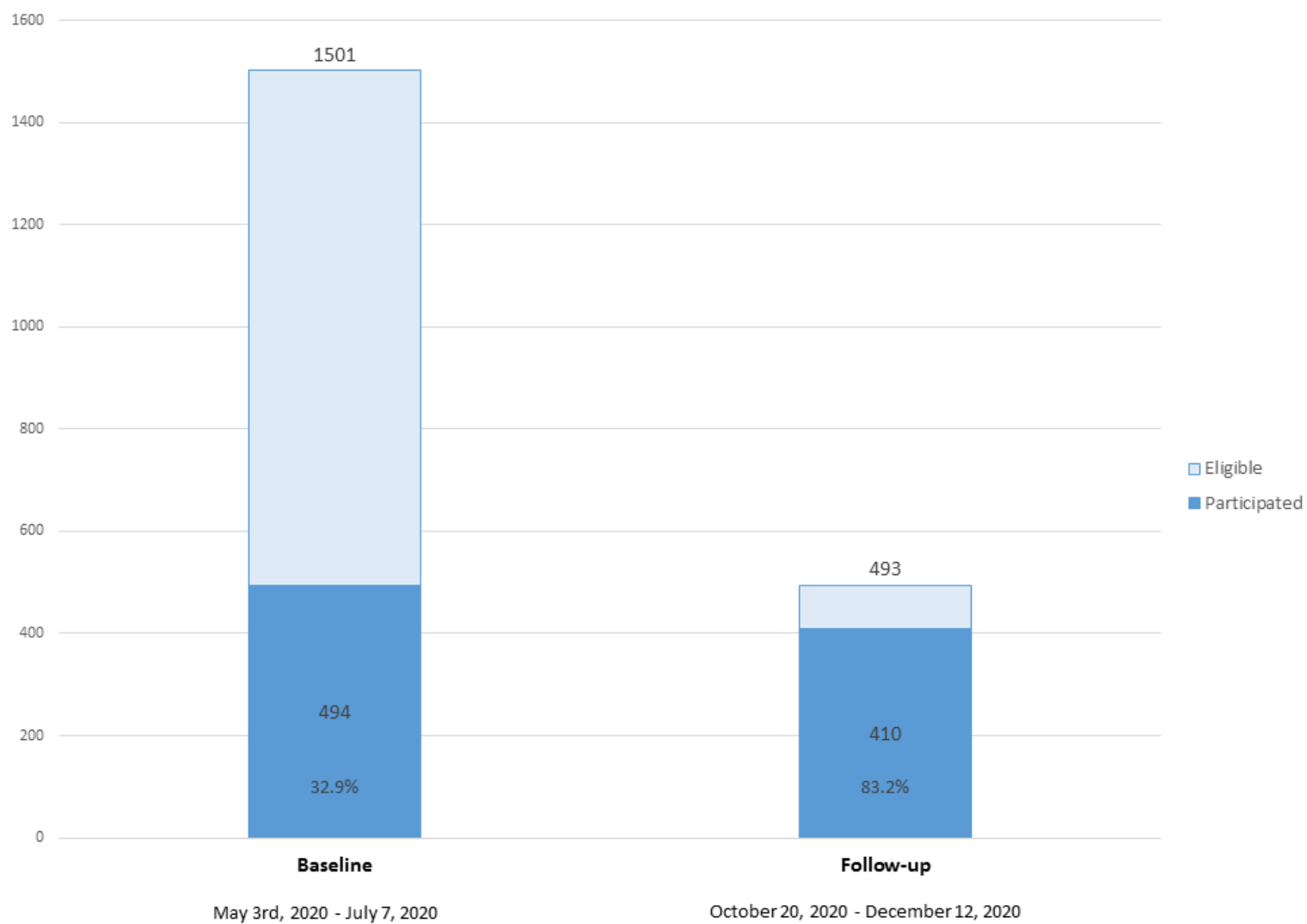

Supplement: Supplementary file 5 [file Image1.pdf]
